# *Saccharomyces boulardii* protects against murine experimental colitis by reshaping the gut microbiome and its metabolic profile

**DOI:** 10.3389/fmicb.2023.1204122

**Published:** 2023-07-10

**Authors:** Hui Gao, Yinzheng Li, Jiqu Xu, Xuezhi Zuo, Tiantian Yue, Huzi Xu, Jie Sun, Meng Wang, Ting Ye, Yan Yu, Ying Yao

**Affiliations:** ^1^Department of Clinical Nutrition, Tongji Hospital, Tongji Medical College, Huazhong University of Science and Technology, Wuhan, China; ^2^Department of Nephrology, Tongji Hospital, Tongji Medical College, Huazhong University of Science and Technology, Wuhan, China; ^3^Department of Nutriology, Oil Crops Research Institute, Chinese Academy of Agricultural Sciences, Wuhan, China; ^4^Hubei Key Laboratory of Lipid Chemistry and Nutrition, Oil Crops Research Institute, Chinese Academy of Agricultural Sciences, Wuhan, China; ^5^Department of Gastroenterology, Tongji Hospital, Tongji Medical College, Huazhong University of Science and Technology, Wuhan, China

**Keywords:** *Saccharomyces boulardii*, ulcerative colitis, gut microbiota, metabolomics, correlation

## Abstract

**Introduction:**

*Saccharomyces boulardii (S. boulardii)* has shown clinical beneficial effect in inflammatory bowel diseases recently. However, the underlying mechanisms remain incompletely understood. The aim of present study was to tested whether *S. boulardii* targets gut microbiota to protect against the development of experimental colitis in mice.

**Methods:**

Female C57BL/6 mice were gavaged with *S. boulardii* for 3 weeks before being challenged with dextran sulphate sodium to induce ulcerative colitis. Bodyweight, diarrhea severity, intestinal permeability, colonic histopathology, colonic inflammatory status, and epithelial cell death of mice were examined. The fecal microbiota and its metabolomic profiles were detected by 16S rDNA sequencing and UPLC-MS, respectively.

**Results and Discussion:**

Supplementation with *S. boulardii* significantly prevented weight loss and colon shortening, lowered colonic inflammation, ameliorated epithelial injury, and enhanced the intestinal barrier integrity in colitis mice. By inhibiting the abundance of pathogenic bacteria and increasing the probiotics abundance, *S. boulardii* improved the microbial diversity and restored the microbiota dysbiosis. Moreover, it also modulated microbial metabolome and altered the relative contents of metabolites involving amino acids, lipids, energy and vitamin metabolisms. These yeast-driven shifts in gut flora and metabolites are were associated with each other and with the inflammation profile in colitis. Collectively, *S. boulardii* exerts protective effects on colitis in mice by reshaping gut microbiome and its metabolic profile, indicating it as a promising therapeutic avenue.

## 1. Introduction

Ulcerative colitis (UC) is a subtype of inflammatory bowel diseases (IBD), characterized as abdominal pain, diarrhea, and blood stool (Hendrickson et al., 2002). In recent years, UC occurs all over the world with a rapidly increasing morbidity due to accelerating circadian rhythm and actuating pressure (Cleynen et al., 2016). Conventional therapies for UC, including aminosalicylate, antibiotics, steroids, immunomodulators and biological applications, have achieved stable management for some patients (Siegel, 2011). Unfortunately, severe adverse reactions like infection and renal injury can not be ignored (Papamichael et al., 2019). It is reported that UC can induce harmful effects on human health, and the relapse-remission cycles and incurable have made it a high-risk factor for colorectal cancer initiation and development (Yamamoto et al., 2020).

Although its aetiology has not been fully elucidated, UC is recognized to be caused by the dysfunction of the intestinal epithelial barrier resulting from dysbacteriosis and chronic inflammation (Martini et al., 2017). In the progression of UC, overreacted inflammatory response eventually leads to tissue injury. Dysbacteriosis is thought to be a vital factor that not merely provokes chronic persistent inflammation (Jackson and Theiss, 2020), but also transforms the intestinal microecology, ultimately triggering metabolic disorders (Li et al., 2017; Bretin et al., 2018). As reported, the composition of intestinal flora in UC patients is generally disturbed, which is characterized by the reduction in diversity and the increase in potential pathogenic bacteria, for instance *Proteobacteria* and adherent/invasive *Escherichia coli* (Zuo and Ng, 2018). Hence, to restore colonic epithelial homeostasis, targeting the gut microbiota may be an alternative candidate with novel therapeutic effects and fewer side effects for UC.

The probiotic *Saccharomyces boulardii* (*S. boulardii*) is a yeast strain of the species *Saccharomyces cerevisae*, which can be reactivated rapidly in the gastrointestinal tract and adaptable to the mammalian gut (Offei et al., 2019). Given the function of maintaining intestinal flora balance and trophic intestinal effects, *S. boulardii* has been extensively researched and applied for the prevention and combate of acute and chronic enterocolopathies, antibiotic-associated diarrhea, traveler’s diarrhea and *Clostridium difficile* infections (Szajewska and Kolodziej, 2015; Sivananthan and Petersen, 2018). Pharmacokinetics studies have shown that oral treatment with a lyophilized preparation of *S. boulardii* produces a stable concentration in the colon within 3 days, while it cannot be detected in feces in 2–5 days after discontinuation (Czerucka et al., 2007). It has shown that the use of *S. boulardii* in the treatment of diarrhea was safe and could reduce the time and frequency of diarrhea (Feizizadeh et al., 2014). Several clinical trials (Thomas et al., 2011; Sivananthan and Petersen, 2018) and experimental studies (Bei et al., 2022; Gu et al., 2022) have also strongly recommended *S. boulardii* as a biotherapeutic agent for the treatment of IBD. Although numerous studies have revealed the efficacy and beneficial effects of *S. boulardii* on certain gastrointestinal diseases, they mainly focused on its therapeutic actions. However, little is known about the preventive actions of *S. boulardii* on colitis and the potential mechanisms behind.

Therefore, this study aimed to clarify the preventive and protective effects of *S. boulardii* on murine experimental colitis induced by dextran sulfate sodium (DSS), and to dissect the underlying mechanism by examining its modulating effects on the gut microbiome and its metabolic profile in addition to inflammatory response.

## 2. Materials and methods

### 2.1. Chemicals

Commercial DSS (molecular weight 36–50 kDa) used for animal model was obtained from MP Biomedicals (Irvine, CA, United States). HPLC grade methanol was bought from Fisher Scientific (Fair Lawn, NJ, United States). The sartorius water purification system (arium®mini, Gottingen, Germany) was applied to gain ultrapure water. Besides, pyridine and chloroform were GC grade from China National Pharmaceutical Group Corporation (Shanghai, China). The preparation of *S. boulardii* (strain number is CICC 1903) was provided by lyophilization from Angel Nutritech Co., Ltd. (Hubei, China. 2,017,081,701), which was suspended in double-distilled water and administered orally by intragastric.

### 2.2. Animals and treatment

All procedures were strictly executed in the light of the international guidelines for the ethical use of laboratory animals and the guidelines of Animal Management Rules of the Ministry of Health of the People’s Republic of China (documentation Number 55, 2001, Ministry of Health of PR China). Moreover, all animal care and experimental procedure were approved by the Animal Care and Use Committee (ACUC) at Tongji Medical College, Huazhong University of Science and Technology, China (S2135).

It is reported that UC commonly affects young females (Ooi et al., 2010). Thus, female C57BL/6 mice, weighing 20 to 24 g (8 weeks old), purchased from Beijing Hua Fukang Laboratory Animal Technology Co., Ltd. (Beijing, China), were maintained in specific pathogen-free (SPF) condition with ambient temperature (25 ± 2°C) and 55% humidity, under a 12:12 h light:dark cycle, Animals were divided into three groups of equal size (*n* = 18) in a randomized allocation after one-week acclimatization: Control (non-colitis) group, DSS (colitis) group and (*Sb* + DSS) group. Distilled water or *S. boulardii* suspension [the dose set at 10^6^ Colony-Forming Units (CFU)/kg/day] was administered orally by intragastric for 21 consecutive days. Experimental colitis was duplicated in mice by adding 2.5% (wt/vol) DSS to drinking water for 7 consecutive days from the 22nd day (Supplementary Figure S1). Mice were housed in a plastic cage with wood shaving, with standard chow and distilled water freely available. We monitored each mouse daily for these indicators such as body weight, presence of hematochezia and the stool consistency. The estimate of weight loss is the variance between the original weight (day 0) and the weight on any given day. The disease activity index (DAI) was calculated in conjunction with scores of body weight loss, diarrhea and blood from the 21th to the 30th day to evaluate the severity of colitis, according to the Cooper method with slightly modified (Cooper et al., 1993). Once sacrificed, blood was gathered directly by cardiac puncture from the inferior vena cava with a heparinized tube. All measurements were performed blindly. The serum, colonic tissue and fecal samples were collected and stored at −80°C for further detection.

### 2.3. Histology assessment

After fixation in 4% paraformaldehyde solution, the distal segments of colon (1–2 cm from anal margin) were prepared and taken for hematoxylin and eosin (H&E) staining once embedded in paraffin wax. Colonic histological damage was assessed based on tissue damage and cell infiltration. Each section was randomly selected with an inverted microscope in six fields (magnification ×200) and blindly evaluated under a light microscope (Olympus, Japan) by two pathologists. The Standard for pathological scoring was shown in Supplementary Table S1.

### 2.4. Circulating cytokines determination

Serum contents of high mobility group box 1 protein (HMGB1), tumor necrosis factor α (TNF-α), interleukin-1β (IL-1β), IL-6, and monocyte chemotactic protein-1 (MCP-1) were thoroughly detected with commercial enzyme-linked immunosorbent assay kits (ELISA) (Neobioscience, China) against the manufacturer’s specifications. All measurements were performed in duplicate.

### 2.5. Immunofluorescence assessment

The colon tissues of mice (6 mice each group) were fixed and permeabilized in methanol at-20°C. Subsequently, the sections were stained at 4°C with primary antibodies ZO-1 (1:100) and Occludin (1:100), next to the incubation with secondary antibody for fluorescent labelling. Nuclei were stained for 10 min with mounting medium containing 4,6-diamidino-2-phenylindole (DAPI) (Roche, Switzerland). Images selected from the same area of tissue section were observed by fluorescence microscopy (Olympus, Japan).

### 2.6. Fecal bacteria and bioinformatics analysis by 16S rRNA gene sequencing

The genomic DNAs were extracted from fecal samples by a commercial DNA stool kit (D4015, Omega, Inc., United States) and quantified by NanoDrop 3,300 fluorospectrometer (Thermo Scientific, Wilmington, DE) as directed. Total DNAs were detected by PCR (LC-Bio Technology Co., Ltd., China) after eluted in 50 μL Elution buffer. For PCR amplification, each amplicon of the 16S rRNA genes V3-V4 region set through the forward primer 338Forward (5’-ACTCCTACGGGAGGCAGCAG-3′) and reverse primer 806Reverse (5’-GGACTACHVGGGTWTCTAAT-3′) with slight modification. Successful amplicons were purified using AMPure XT beads (Beckman Coulter Genomics, Danvers, MA, United States) and quantified by Qubit (Invitrogen, United States). Moreover, assessments of the amplicon library size and quantity were performed on Agilent 2,100 Bioanalyzer (Agilent, United States) using the Library Quantification Kit for Illumina (Kapa Biosciences, Woburn, MA, United States). In addition, PhiX Control library (V3) (Illumina) was combined with the amplicon library (expected at 30%), which was sequenced on 300PE MiSeq. The sequenced reads and operational taxonomic units (OTUs) were applied to assign bacterial taxonomic classification, with a 97% threshold of pairwise identity. The α-diversity was assessed by Mothur to discriminate significantly different species between groups. As to evaluate the β-diversity, several unsupervised multivariate statistical methods including principal coordinate analysis (PCoA), non-metric multidimensional scaling (NMDS) analysis and principal components analysis (PCA) were operated by QIIME2. We deposited the original sequencing data in a NCBI BioProject with the accession number PRJNA639324. To identified differences in biologically relevant taxonomic biomarkers among groups, linear discriminant analysis effect size (LEfSe) was carried out. We further generated abundance-based correlation networks through enriching different group bacterial genus to detect their correlation. Pearson correlation coefficients were calculated to identify bacteria-metabolite correlations. Important relationships were identified as |Rho| ≥ 0.5 and *p*-values <0.05, between the centered log-ratio-transformed bacterial genus and correlative metabolites. Differences in metabolite concentrations between 2 phenotypes were detected by Student *t*-tests, where the cut-off value of VIP > 1.0 screened as statistically significant.

### 2.7. Measurement of fecal metabolites

After precooled with 50% methanol and incubation, approximate 20 μL extraction mixture was gained and preserved overnight at −20°C. All metabolites were collected by the liquid chromatography–tandem mass spectrometry (LC–MS/MS) system followed machine orders. In brief, an ultra-performance liquid chromatography (UPLC) system (SCIEX, United Kingdom) was used to separate all chromatographicions, followed by an ACQUITY UPLC T3 column (100 mm × 2.1 mm, 1.8 μm, Waters, United Kingdom) used for the reversed phase separation. The collected MS data preprocessing were analyzed by a software of XCMS, which identified every ion according to integrating retention time (RT) with m/z data. As a result, outlier detection and batch effects evaluation were conducted through PCA in view of the pre-processed dataset.

### 2.8. Statistical analysis

Data are expressed as means ± SEM. One-way analysis of variance (ANOVA) followed by Dunnett’t test were performed to reveal the differences among groups. Correlation analysis was conducted to achieved the pairwise relationships among the secondary metabolites obtained by metabolomics, the significantly different genera acquired by 16S sequencing analysis, and inflammatory parameters. Statistical analyses were carried out by SPSS 25.0 software (SPSS Inc., Chicago, IL, United States) with *p* < 0.05 served as significance. Difference between metabolites and OTU were conducted in R version 3.5.2 using QIIME2.

## 3. Results

### 3.1. *Saccharomyces boulardii* alleviated DSS-induced colitis in mice

**Figure 1:**
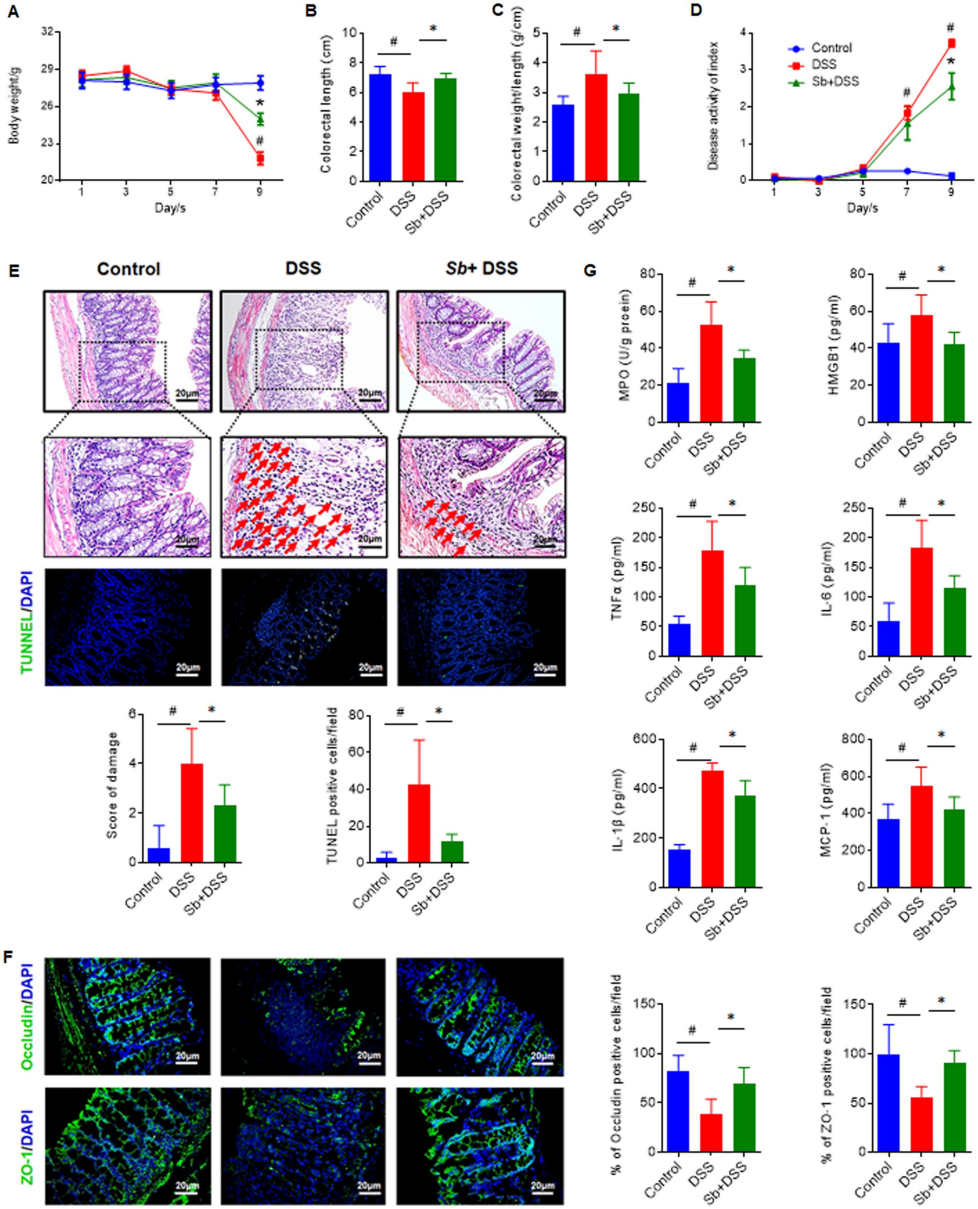
*Saccharomyces boulardii* (*Sb*) alleviated clinical symptoms in DSS-induced colitis. **(A)** Average body weight. **(B)** Colon length of mice. **(C)** Colorectal weight/length ratio of mice. **(D)** DAI were recorded and calculated every other day. **(E)** Appearance of H&E stained colon section and histopathological score. (Magnification: ×200; Scale bar: 50 μm). Immunofluorescence analysis and score of TUNEL (green) in colon mucosa (200 × magnification). DAPI was used for nuclear staining (blue). **(F)** Representative immunofluorescence images and quantification of Occludin and ZO-1, in distal ileum (200 × magnification; Scale bar: 20 μm). DAPI was used for nuclear counterstaining (blue). **(G)** Serum inflammatory mediators of MPO, HMGB1, TNF-α, IL-6, IL-1β and MCP-1 were determined by ELISA kits. Data are presented as mean ± SEM of 6 mice per group. ^#^*p* < 0.05 compared with the control group; ^*^p < 0.05 compared with the DSS group. *Sb*, *S. boulardii*.

Compared to the control, DSS administration induced remarkable reductions in body weight (*p* < 0.05, Figure 1A) and colon length (*p* < 0.05, Figure 1B), caused increased colon weight/length ratio (*p* < 0.05, Figure 1C) and led to higher DAI scores in mice from the seventh day (*p* < 0.05, Figure 1D). Treatment with *S. boulardii* alleviated the colon shortening and body weight loss, lowered the ratio of colon weight/length, and gradually attenuated the elevated DAI scores in DSS-induced colitis mice (all *p* < 0.05). Histological examinations further revealed that in contrast to the healthy colon tissues of control mice, DSS administration caused obvious distortion of crypt epithelium, severe mucosal damage, colonic epithelial cell apoptosis and inflammatory cells infiltration into the mucosa and sub mucosa. However, these were relieved along with *S. boulardii* treatment (*p* < 0.05, Figure 1E). Moreover, the TUNEL assay revealed that *S. boulardii* treatment effectively inhibited cell apoptosis triggered by DSS in colonic tissue, indicating a strong prevention of *S. boulardii* against cell death in colitis mice (*p* < 0.05, Figure 1E).

Given the above histopathological changes, we next evaluated the impact on the intestinal barrier integrity. Compared to control, tight junction components Occludin and ZO-1 were markable reduced in DSS-fed mice revealed by immunofluorescence analysis (both *p* < 0.05, Figure 1F). Whereas *S. boulardii* treatment improved the epithelial integrity by enhancing tight junction proteins in colitis mice induced by DSS.

For the sake of exploring the potential pharmacological effects of *S. boulardii* on inflammation in colitis, MPO, which is a typical inflammatory indicator of colitis reflecting the neutrophil counts, was measured. As depicted in Figure 1G, MPO activity was more obviously in mice administered DSS than the control (*p* < 0.05). Moreover, the serum pro-inflammatory factors including HMGB1, TNF-α, IL-6, IL-1β and MCP-1 were dramatically increased after DSS administration (all *p* < 0.05), while significantly reduced as *S. boulardii* treatment (all *p* < 0.05). Altogether, these results reveal that *S. boulardii* can mitigate the pathogenic symptoms of DSS-induced colitis, suggesting the preventive effects of *S. boulardii* on experimental colitis in mice.

### 3.2. *Saccharomyces boulardii* reshaped the gut microbiome in colitis mice

**Figure 2:**
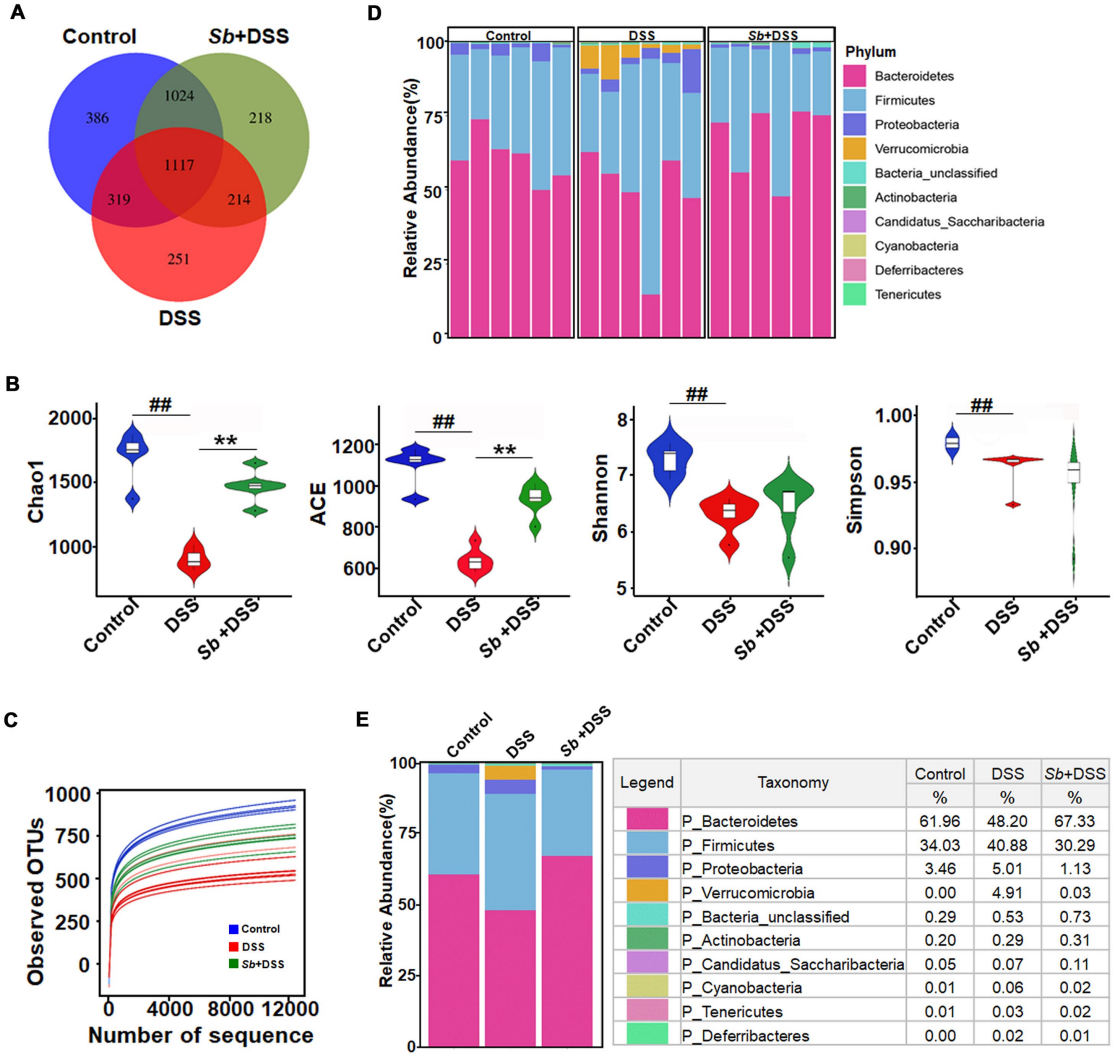
Impact of *S. boulardii* administration on intestinal microbiota in DSS-induced colitis mice. **(A)** A Venn diagram showing the overlap of the OTUs identified in the intestinal microbiota among three groups. **(B)** α-diversity indexes of gut microbiota among three groups. **(C)** The rarefaction curve of the observed OTUs. **(D,E)** Gut microbial pattern at the phylum level within **(D)** and among **(E)** the control, DSS and (*Sb* + DSS) groups were assessed using 16S high throughput sequencing. Blue-, Red-, and green-shaded areas represent the control, DSS and *Sb* + DSS samples, respectively. *n* = 6 per group. ^##^*p* < 0.01 compared with the control group; ^**^p < 0.01 compared with the DSS group. *Sb*, *S. boulardii*.

Overlapping OTU data from the Venn diagram displayed that the control mice existed the highest amount of microbes (2846), whereas this number in DSS-fed mice was minimum (1901). Moreover, *S. boulardii-*treated mice shared more overlapping microbes with the control than mice fed with DSS (Figure 2A). The α-diversity indices of Chao1, ACE, Shannon and Simpson were analyzed utilizing OTU species and abundance to dissect species diversity in samples. In general, Chao1 and ACE proxied for species abundance, meanwhile Shannon and Simpson indicated for community diversity, respectively. As shown in Figure 2B, mice administered DSS displayed a significant reduction in all α-diversity indexes compared to the control animals, while *S. boulardii* treatment increased the community richness but not community diversity in DSS-induced colitis mice. Overall species richness was evaluated through the rarefaction curve of the observed OTUs. Richness in *S. boulardii-*treated colitis mice was close to that in the controls, but higher than that in mice fed with DSS (Figure 2C). The dominant bacterial communities at the phylum level were *Bacteroidetes*, *Firmicutes* and *Proteobacteria* in control mice. However, the gut microbial profile was drastically changed by DSS feeding, with an obvious improvement in the relative abundance of *Firmicutes*, *Verrucomicrobia* and a dramatical diminish in *Bacteroidetes*, resulting in a diminished *Firmicutes/Bacteroidetes* ratio. *S. boulardii* administration prevented the DSS-driven reduction in microbial richness and restored their levels similar to those in control mice (Figures 2D,E).

**Figure 3:**
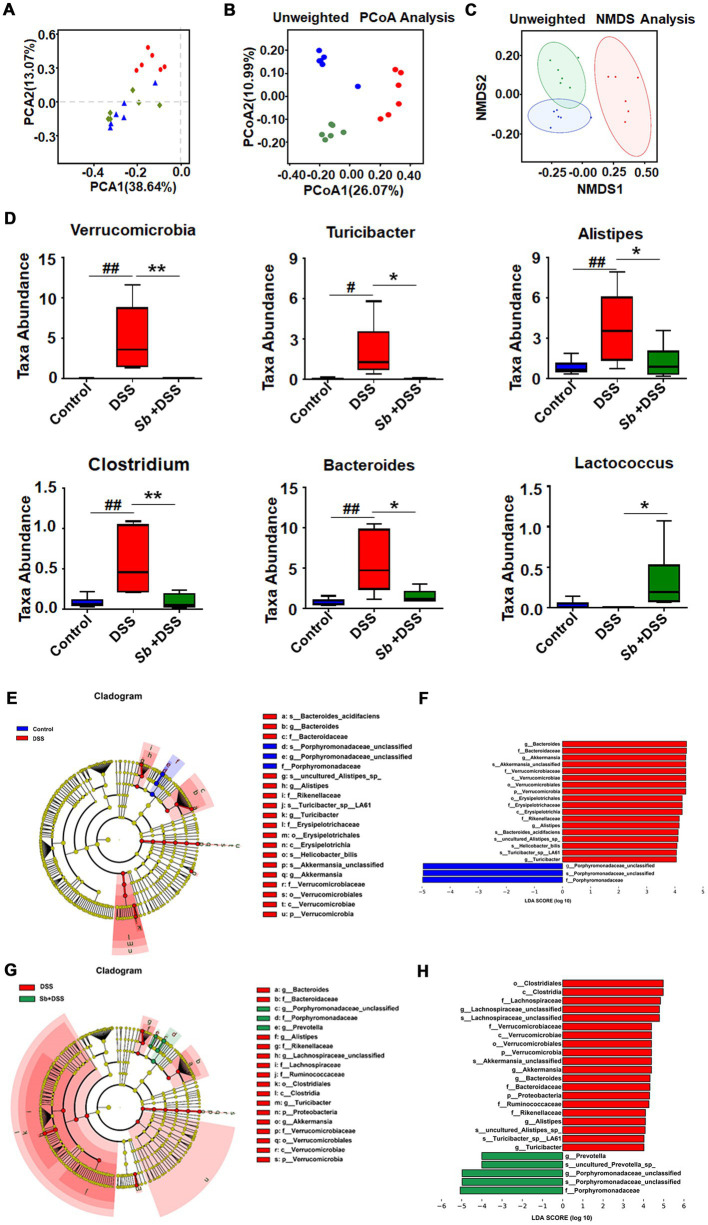
*S. boulardii* transferred the gut microbial community structure toward control profiles. β-diversity of gut microbiota **(A)** PCA, **(B)** PCoA, and **(C)** NMDS analyses based on unweighted UniFrac metrics. **(D)** Top discriminative microbiota among three groups at genus levels as determined by the Metastats analysis. The bottom and top boundaries of each box indicate the 25th and 75th percentiles, respectively; the line within each box represents the median; the bottom and top edges show the minimum and maximum, respectively. **(E–H)** LEfSe and LDA results revealed the significantly differential microbial features at five levels among the three groups. Key bacterial alterations are shown as taxonomic cladogram. Only taxa meeting the linear discriminant analysis (LDA)-significant threshold >4.0 are shown. *n* = 6 per group. ^#^*p* < 0.05 and ^##^*p* < 0.01 compared with the control group; ^*^p < 0.05 and ^**^p < 0.01 compared with the DSS group. *Sb*, *S. boulardii*.

Taking all available sequences into account, comparative analysis revealed a prominently high level in β-diversity (heterogeneity) among groups, suggesting the different variations in response to DSS feeding and *S. boulardii* treatment (Figures 3A–C). Relative abundance performed by Metastats analysis exhibited that enrichment of the *Verrucomicrobia* phylum, and the *Turicibacter*, *Alistipes*, *Clostridium* and *Bacteroides* genera in mice after DSS treatment was the major differences as compared to controls. While *S. boulardii* treatment fully prevented a tendency towards expansion of the microbial communities and, to some extent, restored to the control levels (Figure 3D). Moreover, the LEfSe approach identified *Bacteroides*, *Akkermansia*, *Verrucomicrobia*, *Erysipeiotrichaceae*, *Alistipes*, *Turicibacter*, along with a major decrease in family *Porphyromonadaceae* as the major feature discriminating fecal bacterial communities of DSS-induced colitis mice from that of control animals (Figures 3E,F). Importantly, higher abundance of *Porphyromonadaceae* was observed in *S. boulardii*-treated colitis mice when compared with colitis mice induced by DSS (Figures 3G,H), which in turn, supporting the restorative effect of *S. boulardii* on microbiota dysbiosis.

### 3.3. *Saccharomyces boulardii* altered the metabolic profile of gut microbiome in colitis mice

**Figure 4:**
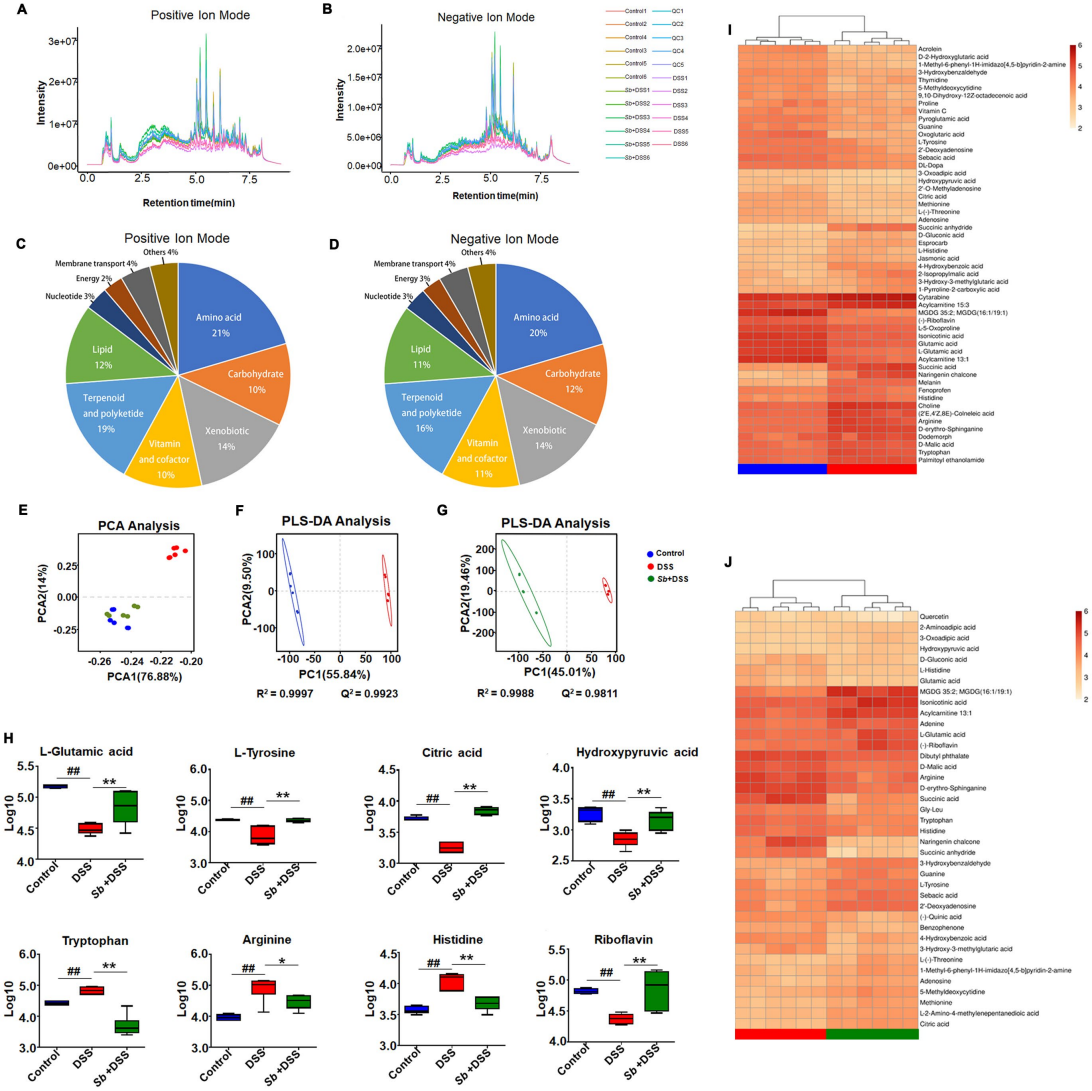
*S. boulardii* altered DSS-induced colitis metabolomic profiles. Total ion current LC–MS chromatogram in positive modern **(A)** and in negative modern **(B)**. Pie chart of the classification of the identified metabolites in positive ion mode **(C)** and negative ion mode **(D)**. PCA score plot of **(C)** samples and QC, **(D)** Control, *Sb* + DSS and DSS groups. **(E)** PCA plot of three groups. **(F)** PLS-DA results for the control group and DSS group. **(G)** PLS-DA results for DSS group and *S. boulardii* + DSS group. Each dot or triangle represents the fecal metabolomic profile of a single sample. **(H)** Box plot visualizations of the relative abundances of the representative metabolites. The bottom and top boundaries of each box indicate the 25th and 75th percentiles, respectively; the horizontal line inside the box is the median; the bottom and top error bars indicate the 10th and 90th percentiles. Heatmap presentation of differential metabolites between **(I)** DSS vs. control, **(J)** *S. boulardii* + DSS vs. DSS groups. *n* = 6 for each group. ^##^*p* < 0.01 compared with the control group; ^*^p < 0.05 and ^**^p < 0.01 compared with the DSS group. *Sb*, *S. boulardii*.

The representative ion current chromatograms of LC–MS disclosed high reproducibility and small retention time drift. Based on chemical ontologies and structure similarity, 436 and 186 peaks were distinguished and assigned to corresponding metabolites in positive and negative ion mode, respectively (Figures 4A,B). A schematic overview of the identified metabolites was classified into nine major groups: amino acid, carbohydrate, xenobiotic, vitamin and cofactor, terpenoid and polyketide, lipid, nucleotide, energy and membrane transport related metabolites (Figures 4C,D).

To describe the dispersion tendency among groups, the PCA was performed and the score plot described an obvious distinction between the colitis mice and the control, similarly with the *S. boulardii* supplementation groups (Figure 4E), indicating striking differences in endogenous metabolites within DSS group and other two groups. It is worth noting that the control and *S. boulardii* group were not fully separated from each other. Subsequently, we employed PLS-DA to further investigate metabolic variations and differential metabolites among groups. Compared to the control, the DSS mice plot showed a markedly variation in the PLS-DA score, characterized by *R*^2^ = 0.9997 and Q^2^ = 0.9923 (Figure 4F). Likewise, a distinctly difference was also observed between the *S. boulardii* supplementation and the DSS groups (*R*^2^ = 0.9988 and Q^2^ = 0.9811, Figure 4G). These plots supplied additional evidence of distinguish in metabolic profiles of DSS-induce colitis and *S. boulardii* treatment.

Specifically, comparison between the DSS-fed mice and the control mice revealed that 58 metabolites were identified as discriminant biomarkers, with 27 enriched and 31 depleted (all *p* < 0.05, Table 1). Moreover, 40 metabolites were significantly changed by *S. boulardii* treatment in contrast to those in the DSS group, with 21 enriched and 19 depleted (all *p* < 0.05). Representative metabolites are visualized in Figure 4H. DSS treatment altered the contents of metabolites involved in the tricarboxylic acid (TCA) cycle and protein metabolism, including _L_-glutamic acid, _L_-tyrosine, citric acid, hydroxypyruvic, tryptophan, arginine and histidine. A hierarchical clustering analysis with selected variant metabolites was run, and the resultant heatmaps presented the relative abundance of discrepant metabolites and relevant metabolic profiles (Figures 4I,J).

**Table 1:** Differential metabolites in DSS group compared with control.

Metabolites	DSS versus Control			*Sb* + DSS versus DSS		
	VIP ^a^	Log_2_ *Fc* ^b^	*p* value ^c^	VIP ^a^	Log_2_ *Fc* ^d^	*p* value ^c^
Acrolein	1.95	−2.63	0.00			
L-(−)-Threonine	1.50	−1.65	0.00	1.55	1.66	0.01
3-Hydroxybenzaldehyde	1.22	−1.48	0.00	2.12	2.33	0.00
Pyroglutamic acid	1.73	−2.01	0.00			
Oxoglutaric acid	2.15	−3.51	0.00			
L-Glutamic acid	1.86	−2.28	0.00	1.05	1.34	0.05
L-2-Amino-4-methylenepentanedioic acid				1.72	1.53	0.00
D-2-Hydroxyglutaric acid	1.70	−2.03	0.00			
3-Oxoadipic acid	1.24	−1.22	0.00	1.41	1.31	0.00
2-Aminoadipic acid				1.34	1.03	0.00
Hydroxypyruvic acid	1.29	−1.38	0.00	1.16	1.01	0.03
Vitamin C	1.22	−1.16	0.00			
L-Tyrosine	1.49	−1.50	0.01	1.60	1.46	0.02
Citric acid	1.36	−1.53	0.00	1.91	1.97	0.00
Sebacic acid	1.63	−1.83	0.00	1.49	1.58	0.00
1-Methyl-6-phenyl-1H-imidazo[4,5-b] pyridin-2-amine	1.54	−1.62	0.00	1.37	1.36	0.01
Adenosine	1.17	−1.06	0.01	1.46	1.22	0.01
1-Pyrroline-2-carboxylic acid	1.16	−1.02	0.00			
Proline	1.20	−1.58	0.01			
Isonicotinic acid	1.45	−1.63	0.00	1.57	1.77	0.01
L-5-Oxoproline	1.31	−1.13	0.00			
Glutamic acid	1.59	−1.56	0.00			
Adenine				1.63	1.45	0.00
Methionine	1.21	−1.21	0.00	1.61	1.50	0.00
Guanine	1.37	−1.62	0.00	1.58	1.72	0.00
DL-Dopa	1.22	−1.11	0.01			
5-Methyldeoxycytidine	1.56	−1.69	0.00	1.52	1.51	0.00
Thymidine	1.18	−1.06	0.01	1.97	1.98	0.02
2’-Deoxyadenosine	1.23	−1.25	0.04			
2’-O-Methyladenosine	1.13	−1.16	0.00			
9,10-Dihydroxy-12Z-octadecenoic acid	1.07	−1.09	0.00			
Acylcarnitine 13:1	1.89	−2.28	0.00	1.51	1.64	0.00
(−)-Riboflavin	1.28	−1.46	0.00	1.53	1.82	0.03
MGDG 35:2; MGDG (16:1/19:1)	2.41	−3.97	0.00	2.27	3.46	0.00
Succinic acid	2.39	4.07	0.00	2.75	−4.19	0.00
D-Malic acid	1.12	1.05	0.01	1.53	−1.37	0.00
4-Hydroxybenzoic acid	1.89	2.34	0.00	1.34	−1.35	0.02
L-Histidine	1.37	1.43	0.00	1.54	−1.47	0.00
3-Hydroxy-3-methylglutaric acid	1.29	1.70	0.02	1.40	−1.62	0.02
Gly-Leu				1.53	−1.36	0.01
(−)-Quinic acid				1.49	−1.28	0.01
2-Isopropylmalic acid	1.71	2.38	0.01			
D-Gluconic acid	1.21	1.35	0.00	1.04	−1.03	0.02
Tryptophan	1.48	1.58	0.00	1.50	−1.45	0.00
Naringenin chalcone	2.52	5.06	0.00	2.90	−5.35	0.00
Fenoprofen	1.04	1.10	0.04			
(2′E,4’Z,8E)-Colneleic acid	1.40	1.50	0.00			
Melanin	2.37	3.68	0.00			
Succinic anhydride	2.78	5.32	0.00	2.77	−4.50	0.00
Choline	1.78	1.98	0.00			
1-Pyrroline-2-carboxylic acid	1.29	1.67	0.04			
Glutamic acid	1.35	1.54	0.00	1.51	−1.58	0.00
Histidine	1.55	1.76	0.00	1.14	−1.11	0.02
Arginine	2.32	5.47	0.01	2.34	−3.30	0.02
Benzophenone				1.21	−1.10	0.01
Dibutyl phthalate				1.39	−1.15	0.00
Jasmonic acid	1.12	1.06	0.00			
Cytarabine	1.21	1.07	0.00			
Esprocarb	1.33	1.50	0.00			
Dodemorph	1.14	1.20	0.04			
Palmitoyl ethanolamide	1.32	1.30	0.00			
D-erythro-Sphinganine	1.56	1.89	0.00	1.87	−1.99	0.00
Acylcarnitine 15:3	1.41	1.28	0.00			
Quercetin				1.06	−1.18	0.03

^a,c^ Only metabolites with VIP values > 1.0 and *p* values < 0.05 were considered statistically significant.

^b^ Fc, Fold-change, calculated as the average mass response (area) ratio between the two classes (Log_2_ *Fc* = log_2_ [DSS/Control]). Thus, positive *Fc* values indicate significantly higher levels in DSS relative to control mice, and negative fold change values indicate significantly lower levels in DSS relative to control mice.

^c^ *Fc*, Fold-change, calculated as the average mass response (area) ratio between the two classes (Log_2_ *Fc* = log_2_ [*Sb* + DSS/DSS]). Thus, positive *Fc* values indicate significantly higher levels in *Sb* + DSS relative to DSS mice, and negative fold change values indicate significantly lower levels in *Sb* + DSS relative to DSS mice.

**Figure 5:**
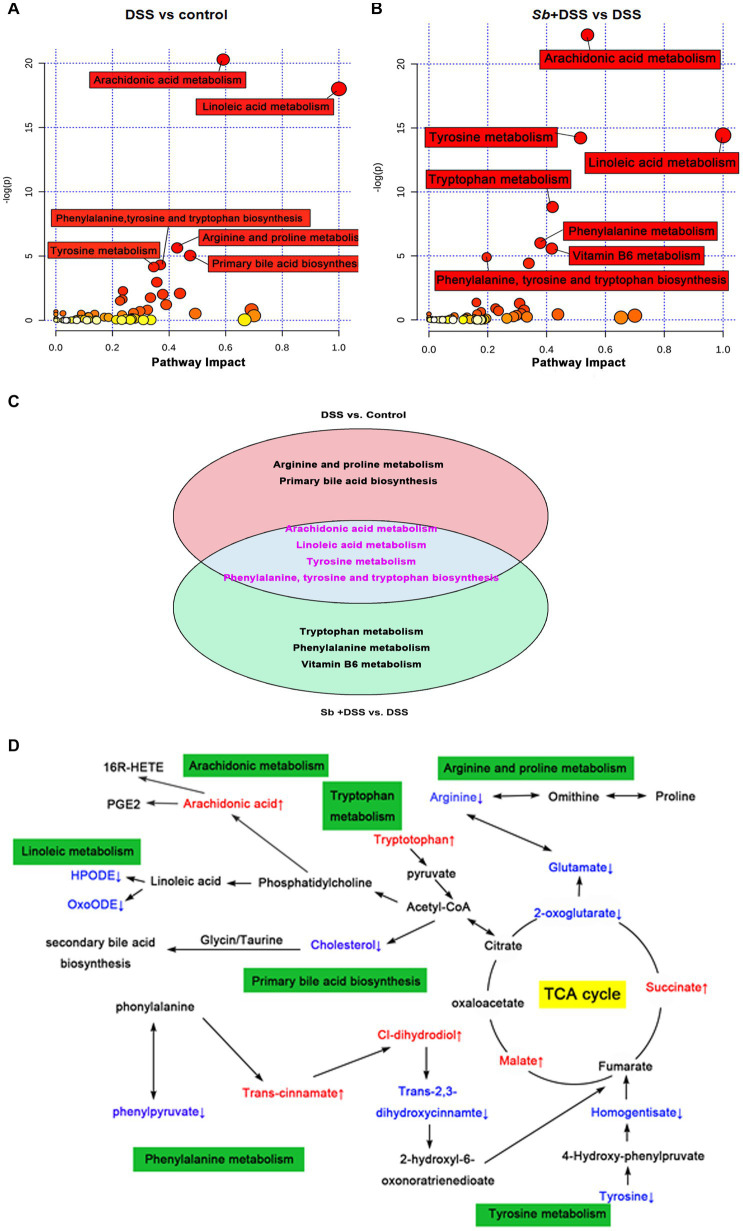
Characterization and functional analysis of key metabolic pathways. Relevant pathway of differential metabolites affected by **(A)** DSS treatment and **(B)** *S. boulardii* treatment. Each bubble represents a metabolic pathway and its size is proportional to the impact of each pathway, with color demonstrating the significance from highest (red) to lowest (white). **(C)** The differentially expressed metabolites in both the comparison groups. The pink circle representing the group of DSS vs. control, and the blue circle representing the group of *S. boulardii* + DSS vs. DSS. The metabolites in the cross area were those identified in both comparison groups. **(D)** Schematic diagram of the metabolomics response to the experimental colitis. Colored boxes represent the key metabolic pathway, red color represents the up-regulation of metabolites, blue color represents the downregulation of metabolites. *n* = 6 for each group. ^##^*p* < 0.01 compared with the control group; ^*^p < 0.05 and ^**^p < 0.01 compared with the DSS group. *Sb*, *S. boulardii*.

Apart from taxonomic composition, differential functional pathways among groups were outlined by MetaboAnaylst based on enrichment and topology analysis (Figures 5A,B). DSS administration affected six pathways, while *S. boulardii* supplementation influenced seven pathways (Supplementary Table S2). Of which, the relevant pathways of synthesis and metabolism, involving arachidonic acid metabolism, linoleic acid metabolism, tyrosine metabolism, phenylalanine, tyrosine and tryptophan biosynthesis, turned out highly consistent (Figure 5C). Based on the calculation results, the final interplay network diagrams among the common metabolite pathways that links with several highly significant biological pathways were also drew up (Figure 5D).

### 3.4. Potential associations among the inflammatory cytokines, gut microbiota and their metabolites in mice

**Figure 6:**
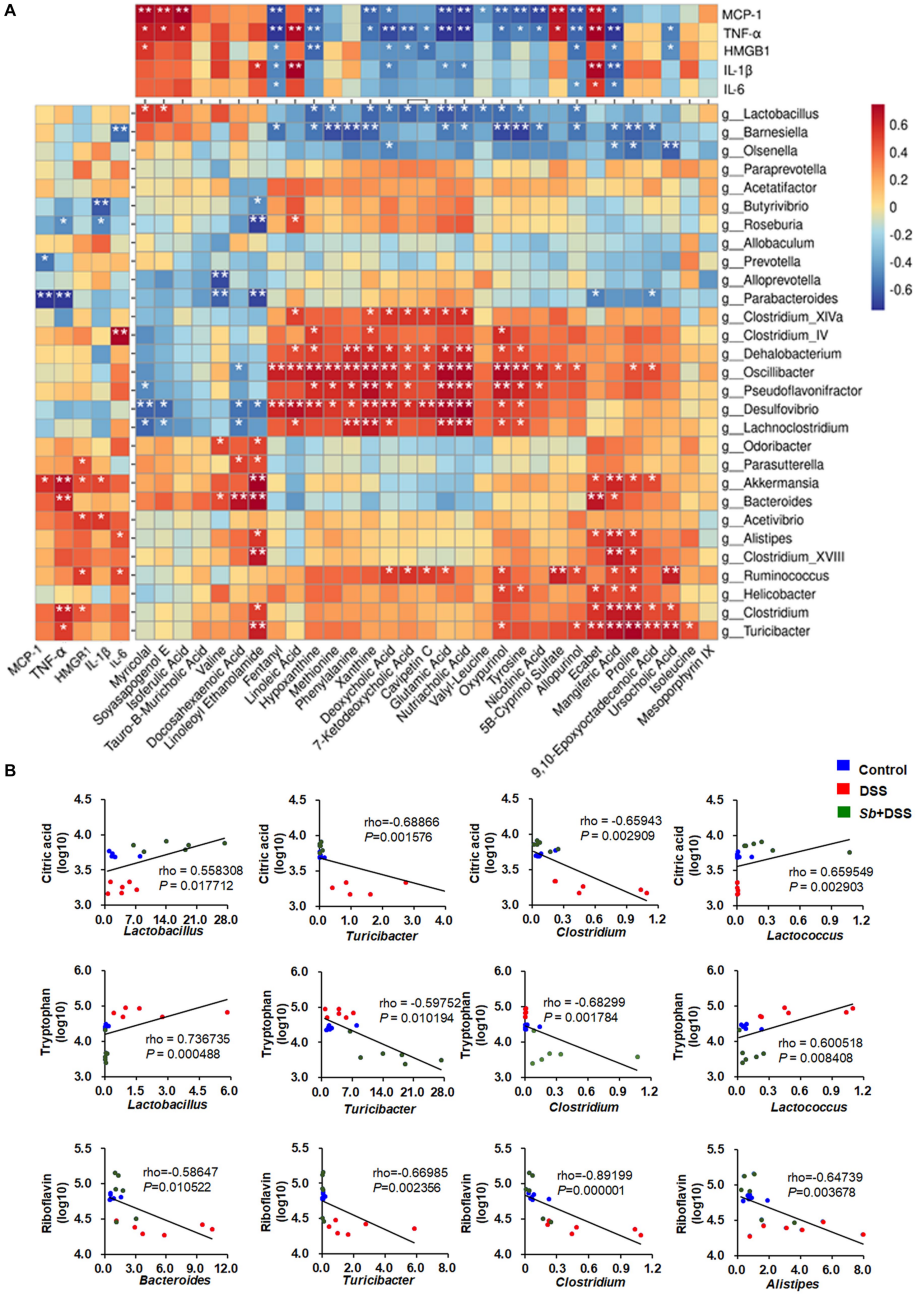
Spearman correlation analyses among the inflammatory cytokines, gut microbial genera and the contents of metabolites. **(A)** Spearman correlation matrix shows the relationship among the inflammatory parameters, intestinal genera and metabolites within the top 30 expression levels. The *rho* values are represented by gradient colors, with red and blue cells representing positive and negative correlations, respectively. An asterisk (*) and two asterisks (**) indicate a significant correlation with *p* < 0.05 and *p* < 0.01, respectively. **(B)** Scatter plots for the correlations between citric acid, tryptophan and *Lactobacillus*, *Turicibacter*, *Clostridium*, *Lactococcus*; and the correlations between riboflavin and *Bacteroides*, *Turicibacter*, *Clostridium, Alistipes* among groups. *n* = 6 for each group. *Sb*, *S. boulardii*.

To better understand the relationships among the inflammatory parameters, gut microbiota and corresponding metabolites in DSS-induced colitis, spearman correlation analysis was carried out. Metabolites were selected according to the intensities within the top 30 expression levels. As shown in Figure 6A, it was found that these 30 gut flora genera appeared either negative or positive association with at least one parameter of inflammation. Thereinto, *Bacteroides*, *Akkermansia*, *Clostridium*, *Alistipes*, *Turicibacter* were positively correlated with parameters that promote colitis, while *Lactobacillus* showed negative correlations. Accordingly, fifteen metabolites (Fentanyl, Hypoxanthine, Xanthine, Deoxycholic acid, 7-Ketodeoxycholic acid, Glutamic acid, Nutriacholic acid, Oxypurinol, Tyrosine, Nicotinic acid, Allopurinol, Mangiferic acid, Ursocholic acid, Cavipetin C, and Valyl-Leucine) were negatively, while seven metabolites (Myricolal, Soyasapogenol E, Isoferulic acid, Linoleoyl ethanol amide, linoleic acid, 5B-Cyprinol sulfate, and Ecabte) were positively correlated with these inflammatory cytokines. In addition, correlations between intestinal flora genera and metabolites were also observed. Wherein, strong correlations were shown in Supplementary Table S3 with |rho| > 0.8 and *p* < 0.05. It’s worth noting that the associations of citric acid with *Turicibacter* and *Clostridium* were negative, but positive with *Lactobacillus* and *Lactococcus*. Tryptophan was negatively associated with *Lactobacillus*, *Lactococcus* and positively correlated with *Turicibacter* and *Clostridium*. Moreover, Riboflavin was negatively correlated with *Bacteroides*, *Turicibacter*, *Clostridium* and *Alistipes* (Figure 6B).

## 4. Discussion

In the present study, *S. boulardii* pretreatment mitigated the classic symptoms of acute colitis in mice, involving up-regulated body weight and colon length, while down-regulated the DAI score. When colitis occurs, immune cells infiltrate into the submucosa and lamina propria, resulting in cryptitis and abscesses, and eventually provoke epithelial degeneration and integrity disruption (Liu et al., 2021). MPO, an important sign of neutrophile granulocytes infiltration (Krawisz et al., 1984), was increased in DSS-induced colitis mice while restored by *S. boulardii* treatment in our study, indicating that *S. boulardii* prevented granulocyte recruitment and inflammatory cells infiltration induced by DSS. This was further reflected in the reduction of colitis-associated serum pro-inflammatory cytokines after *S. boulardii* supplementation. Actually, the reduction of serum inflammatory cytokines is a reasonable target for UC therapy (Moschen et al., 2019). The excessive cell apoptosis in colitis tissue, as a result of hyperinflammatory response, was also alleviated by *S. boulardii* treatment. Another symptom of UC is intestinal epithelial barrier damage, which may further lead to bacterial translocation and other antigens’ entrance (Peng et al., 2019). The tight junction proteins, ZO-1 and occluding, are indicative of the epithelial integrity and junction stability, respectively. Both are generally considered as markers of epithelial barrier function (Peng et al., 2019). Pre-treatment of *S. boulardii* improved the epithelial integrity by enhancing ZO-1 and occludin expression, demonstrating that *S. boulardii* exerts efficient protection and repairment on the intestinal epithelium through various paths (Czerucka and Rampal, 2019). Altogether, these findings underline the prevention and treatment effects of *S. boulardii* on experimental colitis in mice.

Gut microbiota has long been regarded as an important component of intestinal barrier, which is pivotal in the pathogenesis of DSS-induced colitis (Rodriguez-Nogales et al., 2018). Patients with IBD always show microbial community dysbiosis, including decreased microbial diversity and increased detrimental bacteria, compared to healthy individuals (Franzosa et al., 2019). Our results found that DSS-induced colitis mice manifested a lower α-diversity and a distintive β-diversity compared to the control mice. The differences were driven by a reduction in the relative abundance of *Bacteroidetes* and an elevation in *Firmicutes* and *Verrucomicrobia* at the phylum level. This disorder was closely related to experimental colitis in mice, as reflected in previous researches (Peng et al., 2019; Liu et al., 2021). Importantly, after *S. boulardii* treatment, the gut microbiota diversity of colitis mice was improved and the community composition was similar to the control. The protection of *S. boulardii* on gut microbiota dysbiosis have been well reported, but the effects are not completely the same (Szajewska and Kolodziej, 2015; Dong et al., 2019), perhaps due to differences in animal species, the timing of feeding, the treatment regimens, and the baseline microbiota profiles (Liu Z. et al., 2020).

Further evaluation of the gut microbiota composition identified higher abundance of the *Verrucomicrobia* phylum and the *Turicibacter*, *Alistipes*, *Clostridium* and *Bacteroides* genera in colitis mice than in control mice, which are also characteristics of the gut microbiota in IBD patients or mouse models (Liu A. et al., 2020; Parker et al., 2020). However, *S.boulardii* treatment increased the abundance of *Lactococcus*, which has been reported to be positively related to colitis-preventing factors as weight gain and intestinal epithelial barrier intact, whereas negatively associated with colitis-promoting factors as colonic shorten, high DAI and epithelial cells apoptosis (Liu et al., 2019). Especially, *Lactococcus* has been shown to alleviate inflammation via anti-inflammatory activity like stimulating IL-10 production, thereby playing an immunological role in homeostasis of the enterocyte barrier function (Gomes-Santos et al., 2017; Martin et al., 2019). Moreover, oral administration of *S. boulardii* reduced abundance of *Verrucomicrobia*, *Turicibacter*, *Alistipes*, *Clostridium* and *Bacteroides*, which would prevent pro-inflammatory activities and preserve mucus layer integrity, therefore alleviating DSS-induced colitis. The LEfSe analysis further revealed that different dominant bacteria displayed in different groups at OTU level. Particularly, *Porphyromonadaceae* was decrease in DSS-fed mice while served as the most dominant bacteria in *S. boulardii*-treated colitis mice, indicating a restoration of gut microbiota composition. In addition, *Porphyromonadaceae* has been reported conducive to improve the energy availability and regulate the metabolic efficiency via modulating gene expression and stimulating short-chain fatty acids production in colon (Banskota et al., 2017). The implication of these results is that *S. boulardii* alleviates experimental colitis in mice by improving intestinal environment, not merely inhibiting the growth of pathogenic bacteria, but also prompting the probiotics abundance.

To a certain extent, enteric dysbacteriosis may drive metabolism alterations. DSS-induced colitis perturbed the metabolic profiles of the gut microbiome, which was further confirmed by the results of pathway analysis. Meanwhile, the modulations of lipids, amino acids, and energy metabolisms contributed to the remission of *S. boulardii* against colitis. Arachidonic acid is a precursor to eicosanoids and prostaglandin that transduce series of reactions eliciting inflammation and mitochondrial dysfunction (Natarajan et al., 2010). Linoleic acid is able to accelerate fatty acid oxidation, glucose decomposition, and promote the proliferation and differentiation of fat cells (Lalman and Bagley, 2002). Individuals with high linoleic acid in their diet had an increased risk of ulcerative colitis (Tjonneland et al., 2009). Among all the underlining pathways, it is worthy to notice that phenylalanine, tyrosine and tryptophan are aromatic amino acids that play a pivotal role in biological metabolism through synthesizing reductive substances to maintain the balance of cell redox status (Aon et al., 2020). DSS-induced colitis exhibits enhanced oxidative stress level, which is also a feature of the inflammatory environment (Peng et al., 2019), as reflected by decremental biosynthesis of phenylalanine, tyrosine and tryptophan in mice. Meanwhile, the phenylalanine, tyrosine and tryptophan metabolism can fuel to the TCA cycle through pyruvate and acetyl-CoA (Zhang et al., 2018). As a common metabolic pathway, the TCA cycle is essential for energy generation and biosynthetic intermediates in aerobic organisms (Peng et al., 2019). As such, the alterations of phenylalanine, tyrosine and tryptophan biosynthesis and TCA cycle indicate the biosynthesis of compounds for anti-oxidative stress were increased following *S. boulardii* treatment, reflecting a shift toward an inflammation-suppressing microbiome.

More importantly, the specific species of intestinal flora and corresponding metabolites were well correlated, along with the inflammation profile derived by DSS-induced colitis, respectively. The altered gut microbiota and modulation on metabolites, such as citric acid, tryptophan and riboflavin, play a significant role in protein biosynthesis and energy remodelling. In support of these, our data unravel a putative role for *S. boulardii*-dependent metabolites, particularly citric acid and tryptophan, in constraining a range of DSS-induced intestinal dysbacteriosis, while stimulating *Lactobacillus* and *Lactococcus*. This would be in line with previous studies showing negative associations between citric acid, tryptophan and colitis (Agus et al., 2018), as well as *Lactobacillus*, *Lactococcus* and colitis (Oh et al., 2020). *S. boulardii* is a widely used probiotic yeast with promising efficacy in animal experiments and clinical practice. It can produce vigorous acetic acid leading to great antimicrobial and probiotic potency, which may, at least in part, explain the effectiveness in the intestinal tract (Offei et al., 2019). When passing through the intestine, *S. boulardii* interacted with gut microbiota and modified the composition of microbiome, thereby indirectly regulated the host’s immune response (Rodriguez-Nogales et al., 2018). Altogether, these findings imply that *S. boulardii* protects against DSS-induced colitis by modulating metabolism of gut microbiota.

## 5. Conclusion

**Figure 7:**
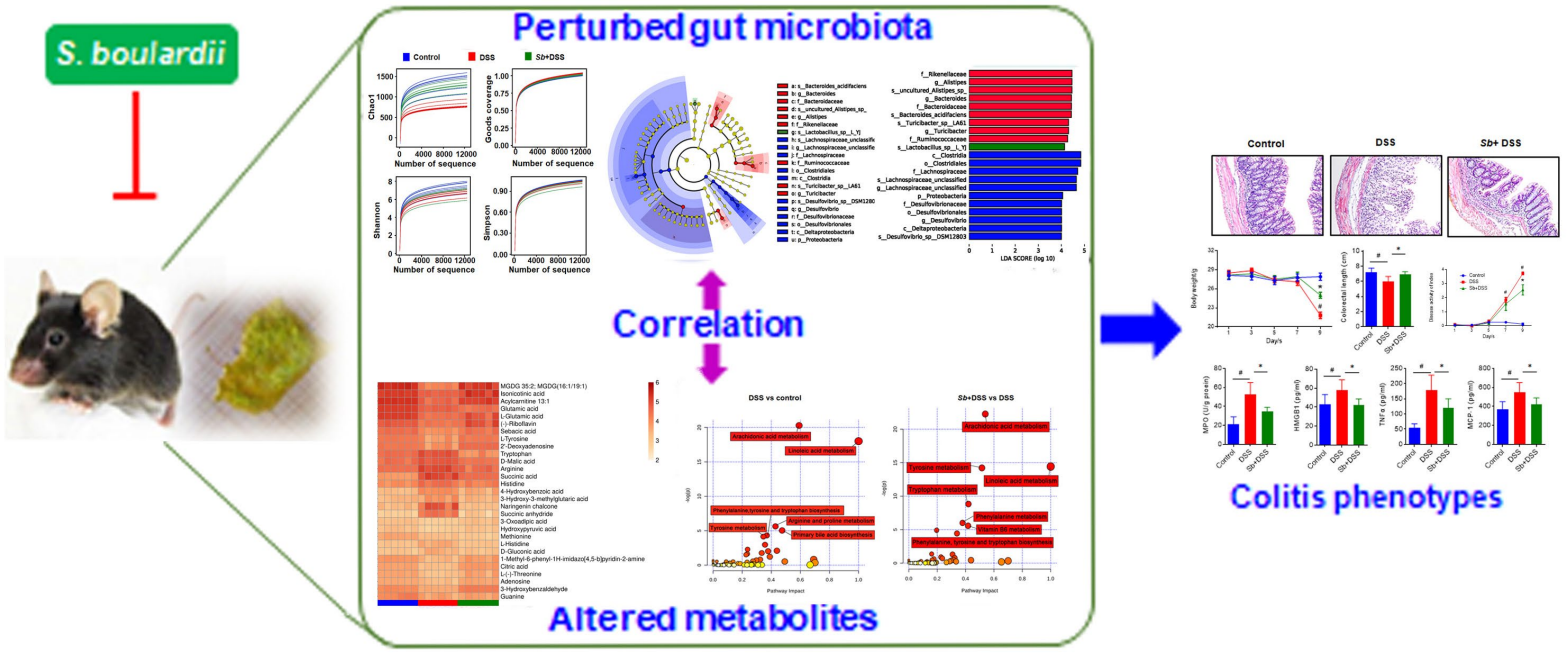
A proposed model for the mechanism of *S. boulardii* against experimental colitis in mice. *S. boulardii* exerts protective effects on experimental colitis in mice by reshaping gut microbiome and its metabolic profile, which are well related to colitis pathology.

Collectively, our study demonstrates that *S. boulardii* exerts protective effects against murine experimental colitis through suppressing systemic inflammation, enhancing tight junction integrity and blocking intestinal epithelial degeneration and necrosis. Using an integrative 16S rRNA sequencing and UPLC-MS analyses, we further show that *S. boulardii* supplementation reshapes the gut microbiome and its metabolic functions in colitis mice. More importantly, *S. boulardii*-drived variations in gut microflora and metabolites are associated with inflammation profile. Our findings indicate that modulation of gut microbiome and its metabolic functions may be the crucial mechanism of action underlying the preventive effects of *S. boulardii* action on experimental colitis in mice (Figure 7). However, further study is merited to clarify the precise mechanisms of action of these dominant microbiota and metabolites.

## Data availability statement

The datasets presented in this study can be found in online repositories. The names of the repository/repositories and accession number(s) can be found in the article/Supplementary material.

## Ethics statement

The animal study was reviewed and approved by the Animal Care and Use Committee (ACUC) at Tongji Medical College, Huazhong University of Science and Technology, China (protocol code: 2135 and date of approval: February 2018).

## Author contributions

HG and YYa contributed to conception and design of the study. HG, YL, JX, and TYu performed the experiments. HX and JS organized the database. MW, TYe and YYu performed the statistical analysis. HG wrote the first draft of the manuscript. HG, YL, and JX wrote sections of the manuscript. All authors contributed to the article and approved the submitted version.

## Funding

This work was supported by the National Natural Science Foundation of China (Grant no. 81703215), Tongji Hospital (HUST) Foundation for Excellent Young Scientist (Grant no. 2020YQ19), Tongji Hospital (HUST) Foundation (Grant no.2022A04) and the Angel Nutritech Nutrition Fund (Grant no. AF2016002).

## Conflict of interest

The authors declare that the research was conducted in the absence of any commercial or financial relationships that could be construed as a potential conflict of interest.

## Publisher’s note

All claims expressed in this article are solely those of the authors and do not necessarily represent those of their affiliated organizations, or those of the publisher, the editors and the reviewers. Any product that may be evaluated in this article, or claim that may be made by its manufacturer, is not guaranteed or endorsed by the publisher.
