# Supplementary material for: Saccharomyces boulardii protects against murine experimental colitis by reshaping the gut microbiome and its metabolic profile
Source: Front Microbiol. 2023 Jul 10;14:1204122. doi: 10.3389/fmicb.2023.1204122 (PMC10363984; doi:10.3389/fmicb.2023.1204122)
Supplement: Supplementary file 1 [file Data_Sheet_1.docx]

Supplementary Material

*Saccharomyces boulardii* protects against murine experimental colitis by reshaping the gut microbiome and its metabolic profile

Hui Gao, Yinzheng Li, Jiqu Xu, Xuezhi Zuo, Tiantian Yue, Huzi Xu, Jie Sun, Meng Wang, Ting Ye, Yan Yu, Ying Yao^*^

*** Correspondence:** Ying Yao, yaoyingkk@126.com

## Supplementary Figures


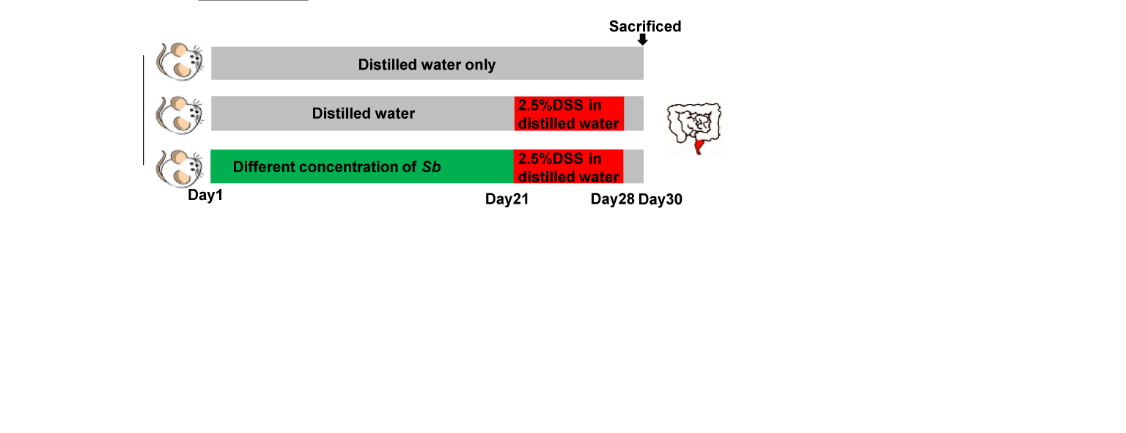


**Supplementary Figure S1.** **Specific processing procedures for experimental animals.** Mice were continuously administrated with 2.5% DSS for 7 days with or without *S. boulardii* (10^5^ and 10^7^ CFU/ml) pretreatment for 21 days. *Sb*, *S. boulardii*.

## Supplementary Tables

**Supplementary Table S1.** **Evaluation of pathological score**

| Score | Epithelial cells | Inflammatory cell infiltration |
| --- | --- | --- |
| 0 | Normal form | No infiltration |
| 1 | Goblet cell loss | Infiltration in basal layer of crypt |
| 2 | Crypt cells loss | Infiltration reaches the mucosal muscle layer |
| 3 | Large area loss of crypt cells | Infiltration to the submucosa |

**Supplementary Table S2. Metabolic pathway analysis among groups**

| Pathway name | Total^a^ | Hits^b^ | Raw *P*^c^ | -log(*P*) | Impact^d^ |
| --- | --- | --- | --- | --- | --- |
| DSS *vs* Control | | | | | |
| Linoleic acid metabolism | 15 | 13 | 1.35E-08 | 1.81E+01 | 1.00 |
| Arachidonic acid metabolism | 62 | 32 | 1.21E-09 | 2.05E+01 | 0.59 |
| Primary bile acid biosynthesis | 47 | 16 | 5.91E-03 | 5.13 | 0.47 |
| Arginine and proline metabolism | 77 | 24 | 3.16E-03 | 5.76 | 0.43 |
| Tyrosine metabolism | 76 | 22 | 1.20E-02 | 4.42 | 0.37 |
| Phenylalanine, tyrosine and tryptophan biosynthesis | 27 | 10 | 1.48E-02 | 4.21 | 0.35 |
| *S. boulardii* *vs* DSS | | | | | |
| Linoleic acid metabolism | 15 | 12 | 5.44E-07 | 1.44E+01 | 1.00 |
| Arachidonic acid metabolism | 62 | 34 | 2.13E-10 | 2.23E+01 | 0.54 |
| Tyrosine metabolism | 76 | 33 | 6.68E-07 | 1.42E+01 | 0.52 |
| Tryptophan metabolism | 79 | 29 | 1.47E-04 | 8.82 | 0.42 |
| Vitamin B6 metabolism | 32 | 13 | 3.80E-03 | 5.57 | 0.42 |
| Phenylalanine metabolism | 45 | 17 | 2.47E-03 | 6.00 | 0.38 |
| Phenylalanine, tyrosine and tryptophan biosynthesis | 27 | 11 | 7.43E-03 | 4.90 | 0.20 |

^a^ Total is the amount of compounds involved in the pathway.

^b^ Hits is the actual matched number from the user uploaded data.

^c^ The raw *P* is the original *P* value calculated from the enrichment analysis.

^d^ Impact value is calculated from pathway topology analysis for comparison among different pathways.

**Supplementary Table** **S3.** **Genera and metabolites with strong correlation**

| Genera | Metabolites | rho | *P-*value | Relation |
| --- | --- | --- | --- | --- |
| *Akkermansia* | Aspartate | -0.81 | 0.00 | negative |
| *Akkermansia* | L-5-Oxoproline | -0.85 | 0.00 | negative |
| *Akkermansia* | XI-2,3-Dihydro-3-Methylfuran | -0.82 | 0.00 | negative |
| *Akkermansia* | Pyroglutamic Acid | -0.80 | 0.00 | negative |
| *Akkermansia* | Ketovaline | -0.81 | 0.00 | negative |
| *Turicibacter* | L-Proline | -0.84 | 0.00 | negative |
| *Turicibacter* | Cinnamaldehyde | -0.85 | 0.00 | negative |
| *Turicibacter* | Methylsuccinic Acid | -0.81 | 0.00 | negative |
| *Turicibacter* | Thymine | -0.82 | 0.00 | negative |
| *Turicibacter* | Nicotinic Acid | -0.83 | 0.00 | negative |
| *Turicibacter* | Styrene | -0.84 | 0.00 | negative |
| *Clostridium-XVIII* | Uracil | -0.83 | 0.00 | negative |
| *Clostridium* | Nicotinic Acid | -0.80 | 0.00 | negative |
